# Alfalfa *MsATG13* Confers Cold Stress Tolerance to Plants by Promoting Autophagy

**DOI:** 10.3390/ijms241512033

**Published:** 2023-07-27

**Authors:** Weidi Zhao, Jiayi Song, Meijia Wang, Xiuxiu Chen, Binghao Du, Yimin An, Lishuang Zhang, Dan Wang, Changhong Guo

**Affiliations:** Key Laboratory of Molecular Cytogenetics and Genetic Breeding of Heilongjiang Province, College of Life Science and Technology, Harbin Normal University, No. 1 of Shida Road, Limin Development Zone, Harbin 150025, China

**Keywords:** *MsATG13*, *Medicago sativa*, autophagy, cold stress, ROS

## Abstract

Autophagy is a conserved cellular process that functions in the maintenance of physiological and metabolic balance. It has previously been demonstrated to improve plant tolerance to abiotic stress. Numerous autophagy–related genes (ATGs) that regulate abiotic stress have been identified, but there have been few functional studies showing how ATGs confer cold stress tolerance. The cold transcriptome data of the crown buds that experienced overwintering of the alfalfa (*Medicago sativa* L.) showed that *MsATG13* is upregulated in response to cold stress. In the present study, we found that *MsATG13* transgenic tobacco enhanced cold tolerance compared to wild–type (WT) plants. Transmission electron microscopy demonstrated that transgenic tobacco overexpressing *MsATG13* formed more autophagosomes than WT plants in response to cold stress conditions. The transgenic tobacco increased autophagy levels due to upregulation of other ATGs that were necessary for autophagosome production under cold stress conditions. *MsATG13* transgenic tobacco also increased the proline contents and antioxidant enzyme activities, enhancing the antioxidant defense capabilities under cold stress conditions. Furthermore, *MsATG13* overexpression decreased levels of superoxide anion radicals and hydrogen peroxide under cold stress conditions. These findings demonstrate the role of *MsATG13* in enhancing plant cold tolerance through modulation of autophagy and antioxidant levels.

## 1. Introduction

Cold is a common abiotic stress condition that can seriously damage crops, inhibiting their growth and yield [1]. It is also a major environmental factor that limits the geographic range of key crops [2]. Cold stress typically causes adverse effects such as seedling stunting, chlorosis, reduced leaf expansion, wilting, and water loss [3]. Furthermore, it induces accumulation of reactive oxygen species (ROS) [4]. High levels of ROS can cause oxidative stress [5,6], which disrupts membrane systems and organelles, causes protein damage and aggregation, and ultimately leads to disordered plant physiology and metabolism [7]. To prevent such oxidative stress, plant cells must have the capacity to quickly remove excess ROS to maintain normal physiological and metabolic balance. Enzymatic and non–enzymatic systems are important methods for plants to remove excess ROS [8]. The primary antioxidant enzymes are catalase (CAT), peroxidase (POD), and superoxide dismutase (SOD) [9]. The non–enzymatic ROS protection system includes the osmotic protector proline [10]. Both systems are critical in preventing ROS–induced damage, and thus promote stress tolerance.

Oxidative stress can also induce autophagy [11,12], which contributes to the regulation of physiological and metabolic balance through autophagosomes in plant cells [13,14]. Previous studies have identified that autophagy–related genes (ATGs) play important roles in the key steps of autophagosome formation: initiation, nucleation, membrane extension, and maturation. The genes that control these steps therefore regulate an organism’s capacity for autophagy, thereby regulating abiotic stress tolerance. For example, in an apple (*Malus domestica*), overexpression of *MdATG5* enhances autophagy levels; this may regulate antioxidant enzyme activity and promote antioxidant accumulation, improving plant defense capabilities [15]. Overexpression of *MdATG10* in an apple also enhances autophagy levels, which has been shown to improve salt tolerance, potentially by improving ion homeostasis [16]. *Arabidopsis thaliana* plants overexpressing *MaATG8* have higher endogenous abscisic acid (ABA) levels, autophagy levels, and drought tolerance than wild–type (WT) plants, which is likely due to the association between ABA biosynthesis and autophagy [17]. In pepper (*Capsicum annuum*) and wheat (*Triticum aestivum*), cold stress promotes autophagosome formation and increases expression levels of *CaATG13* and *TaATG8*, respectively [18,19]. Although several studies have demonstrated that ATGs are induced by cold stress, there have been no reports detailing the function of ATGs in terms of plant tolerance to cold stress.

Alfalfa (*Medicago sativa* L.) is a type of perennial forage legume that is cultivated throughout the world [20]. It has such excellent quality and palatability that it has been referred to as the “king of forage” [21]. However, overwintering is a key problem that restricts its sustainable growth and utilization. *M. sativa* cv. ‘Zhaodong’ is a native cultivar of alfalfa found in Heilongjiang Province, China, that has strong cold resistance; the survival rate of overwintering *M. sativa* ‘Zhaodong’ remains above 90%, even with temperatures below −30 °C [22]. Previous transcriptomic data from *M. sativa* ‘Zhaodong’ (accession number SRP060503) showed that a gene related to autophagy, *MsATG13*, was significantly upregulated under cold stress. *ATG13* plays a primary controlling role in the initiation process of induced autophagy. When plant cells sense stress stimuli, *ATG13* undergoes dephosphorylation and associates with *ATG1*. The formation of the *ATG13–ATG1* complex activates the kinase activity of *ATG1*, initiating the recruitment of other autophagy-related proteins to collectively form the pre-autophagosomal structures and participate in the initiation of autophagosome formation [23,24,25]. To determine the function of *MsATG13* in plant responses to cold stress, we here isolated *MsATG13* from *M. sativa* ‘Zhaodong’ and analyzed its expression patterns in response to cold stress. We also quantified parameters associated with stress responses, including autophagosome formation and antioxidant levels, among WT plants and transgenic tobacco overexpressing *MsATG13*. The results revealed the effects of *MsATG13*–mediated autophagy on the antioxidant system and cold tolerance in transgenic tobacco. This study not only demonstrates the functionality of a key *ATG* in cold tolerance but also provides a theoretical basis for further discovery and characterization of cold tolerance genes in alfalfa.

## 2. Results

### 2.1. Characterization and Expression Profiles of MsATG13 

**Figure 1:**
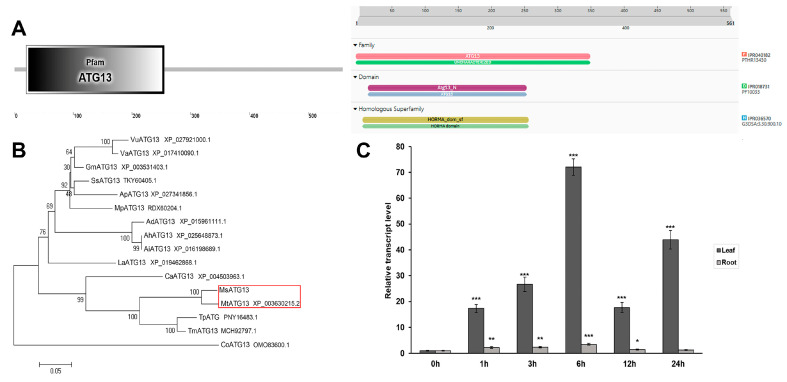
Characterization of *MsATG13*. (**A**) Prediction of the functional domain present in MsATG13. (**B**) Phylogenetic analysis of MsATG13 and other family members. MsATG13 (*Medicago sativa* L., UUH54286.1); AdATG13 (*Arachis duranensis*, XP_015961111.1); AhATG13 (*Arachis hypogaea*, XP_025648873.1); AiATG13 (*Arachis ipaensis*, XP_016198689.1); ApATG13 (*Abrus precatorius*, XP_027341856.1); CaATG13 (*Cicer arietinum*, XP_004503963.1); CoATG13 (*Corchorus olitorius*, OMO83600.1); GmATG13 (*Glycine max*, XP_003531403.1); LaATG13 (*Lupinus angustifolius*, XP_019462868.1); MpATG13 (*Mucuna pruriens*, RDX60204.1); MtATG13 (*Medicago truncatula*, XP_003630215.2); SsATG13 (*Spatholobus suberectus*, TKY60405.1); TmATG13 (*Trifolium medium*, MCH92797.1); TpATG (*Trifolium pratense*, PNY16483.1); VaATG13 (*Vigna angularis*, XP_017410090.1); and VuATG13 (*Vigna unguiculata*, XP_027921000.1). Red square represented the member with the highest sequence identity to the MsATG13. (**C**) Relative *MsATG13* expression levels in alfalfa leaves and roots in response to cold stress (4 °C). All data were normalized to the expression level in the control (0 h). Data are presented as the mean ± standard deviation from three biological replicates, the *MsActin2* (JQ028730.1) was used as the internal control gene. Asterisks indicate significant differences (* *p* < 0.05, ** *p* < 0.01, *** *p* < 0.001) compared to 0 h (Student’s *t*-test).

*MsATG13* comprised a 1686–bp ORF encoding a predicted protein of 561 amino acids in length. Conserved protein domain prediction suggested that it contained an ATG13 domain, which is typical of ATG13 family members. The InterPro search results indicated an ATG13 domain (PF10033) where the amino acids located at positions 20–253 of MsATG13 belonged to the HORMA domain (Figure 1A). Members of theATG13 family are involved in cytoplasm to vacuole transport (Cvt), and more specifically in Cvt vesicle formation. They are probably involved in the switching machinery regulating the conversion between the Cvt pathway and autophagy [26,27,28]. Additionally, phylogenetic analysis showed that MsATG13 was closely related to ATG13 in several species of the family Leguminosae, including *Medicago truncatula*, *Trifolium pratense*, and *Trifolium medium*. MsATG13 shared the highest sequence identity (94.75%) with MtATG13 (Figure 1B).

Analysis of control and cold–stressed *M. sativa* plants demonstrated that *MsATG13* was upregulated by cold stress in both the leaves and the roots, although relative expression levels were significantly higher in the leaves than in the roots. *MsATG13* expression peaked twice in the leaves, first at 6 h (by 72.1–fold compared to 0 h), then at 24 h (by 43.9–fold). In the roots, *MsATG13* expression reached a maximum (3.4–fold higher than 0 h) at 6 h of cold stress (Figure 1C).

### 2.2. MsATG13 Overexpression Conferred Cold Stress Tolerance

**Figure 2:**
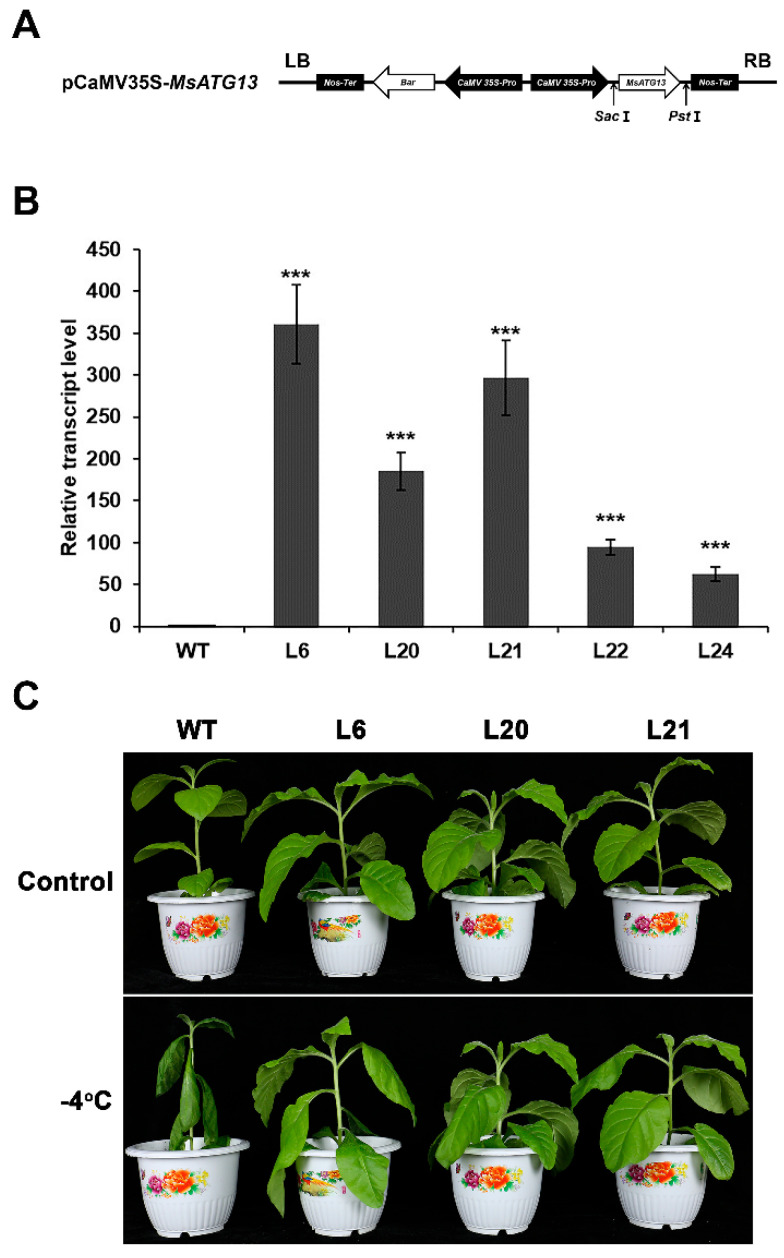
Evaluation of cold tolerance in transgenic tobacco. (**A**) Structure of the *MsATG13* overexpression vector pCaMV35S–*MsATG13*. (**B**) Relative *MsATG13* expression levels in transgenic tobacco lines. Expression levels were normalized to that in WT plant. Data are presented as the mean ± standard deviation from three biological replicates, the *NtGAPDH* (XM_016655379.1) was used as the internal control gene. Asterisks indicate significant differences (*** *p* < 0.001) compared to WT plants (Student’s *t*–test). (**C**) Phenotype of WT and transgenic tobacco plants in the control (upper panel) and cold–treatment (lower panel) groups.

Tobacco plants were transformed with the recombinant overexpression vector pCaMV35S–*MsATG13* containing a screening gene with glufosinate–ammonium resistance (Figure 2A) to determine how *MsATG13* would affect cold tolerance. Out of 24 putative transformants, five transgenic lines were positively identified. Three lines (L6, L20, and L21) were selected for further analysis because they showed higher transcript levels of *MsATG13* expression levels (Figure 2B). We then compared the morphological and physiological differences of the WT and transgenic lines. There were no phenotypic differences between the WT and transgenic plants grown at 25 °C. After cold stress, WT plants were severely wilted, whereas there was no visible damage to transgenic plants (Figure 2C). This suggested that *MsATG13* conferred resistance to cold stress.

### 2.3. MsATG13 Overexpression Increased Autophagosome Formation

**Figure 3:**
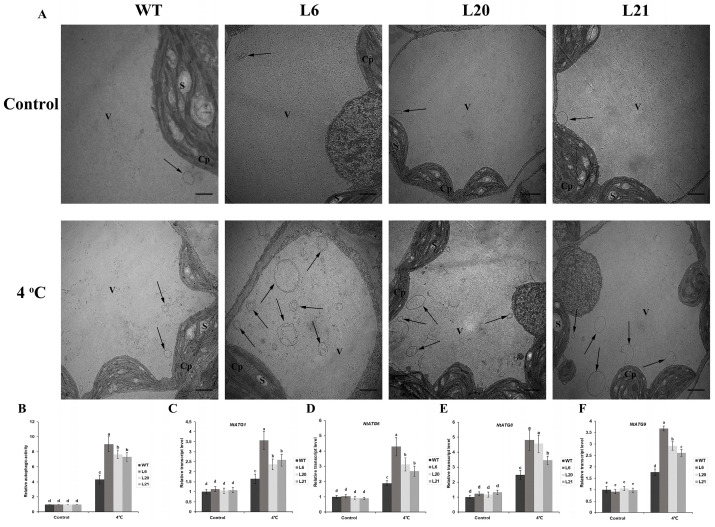
Analysis of autophagy in WT and *MsATG13* overexpressing tobacco plants. (**A**) Representative images of leaf cells in tobacco plants incubated at 4 °C. Cp, chloroplast; V, vacuole; S, starch. Autophagosomes are indicated with black arrows. Scale bar = 1 µm. (**B**) Relative autophagic activity normalized to the activity of transgenic tobacco lines or WT was shown in (**A**). More than 10 cells were used for statistics. (**C**–**F**) Expression levels of (**C**) *NtATG1*, (**D**) *NtATG6*, (**E**) *NtATG8*, and (**F**) *NtATG9* in WT and transgenic tobacco plants. All data were normalized to the expression level in WT of the controls, respectively. Data are presented as the mean ± standard deviation from three biological replicates, the *NtGAPDH* (XM_016655379.1) was used as the internal control gene. Letters above each bar indicate statistical significance groups at *p* < 0.05 (one–way analysis of variance).

To analyze potential differences in autophagosome formation between the WT and transgenic plants, we observed and quantified autophagosomes via TEM. Autophagosomes, which are double–membraned vesicles, sequester and eliminate damaged cellular components by fusing with the vacuole [29]. Degradation occurs after autophagosomes fuse with the vacuole, and the products are exported into the cytoplasm for recycling [30,31]. There were relatively few autophagosomes in any of the lines under control conditions. However, cold-treated transgenic plants had approximately 1.7–2.1 times more autophagosomes than cold-treated WT plants (Figure 3A,B). To determine whether the differences in autophagosome number corresponded to differences in autophagic function, we measured the expression levels of other key ATGs. Under control conditions, there were no significant differences (remained around 1–fold normalized to WT of the controls, respectively) in the expression of *NtATG1*, *NtATG6*, *NtATG8*, and *NtATG9* between transgenic and WT plants. Both the WT and the transgenic plants showed upregulation of all four ATGs after cold stress, but the expression of four NtATGs in transgenic tobacco were significantly higher than those in WT (Figure 3C–F). These results indicated that *MsATG13* promoted autophagosome formation and function in tobacco.

### 2.4. MsATG13 Reduced Cold–Induced Oxidative Damage

**Figure 4:**
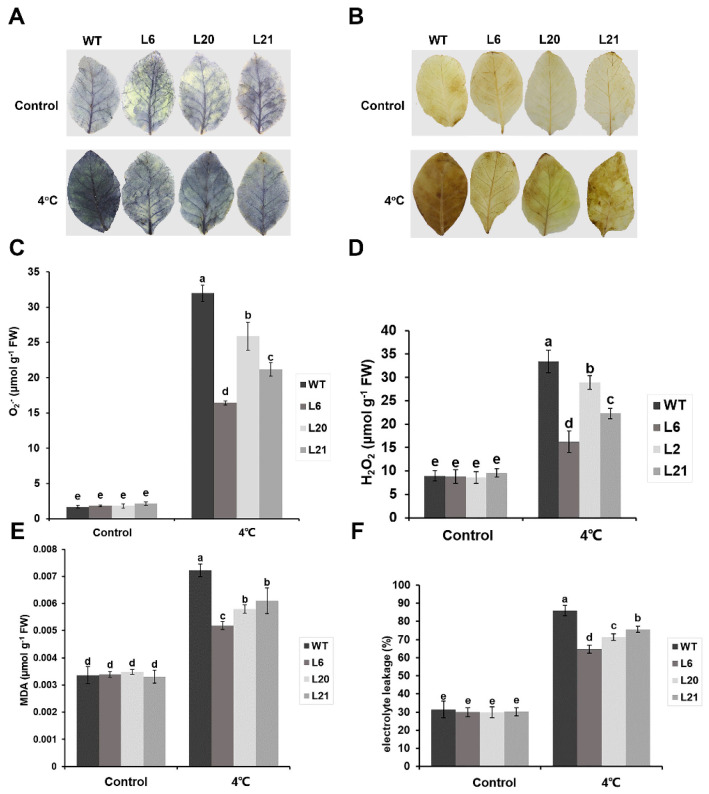
Evaluation of oxidative damage in transgenic tobacco. (**A**,**B**) The leaves of WT and transgenic tobacco plants overexpressing *MsATG13* incubated at 4 °C were stained with (**A**) p–Nitro blue tetrazolium chloride (NBT) and (**B**) diaminobenzidine (DAB). (**C**,**D**) Quantification of (**C**) O_2_^−^ and (**D**) H_2_O_2_ levels in the leaves of WT and transgenic tobacco plants incubated at 4 °C. (**E**,**F**) Quantification of (**E**) malondialdehyde (MDA) and (**F**) electrolyte leakage in the leaves of WT and transgenic tobacco plants incubated at 4 °C. Data are presented as the mean ± standard deviation from three biological replicates. Letters above each bar indicate statistical significance groups at *p* < 0.05 (oneway analysis of variance).

Plant cold stress responses generally include ROS accumulation, which can result in oxidative damage to the cells. To determine whether ROS accumulation was affected by *MsATG13* expression, we performed histochemical staining for O_2_^−^ and H_2_O_2_. For both ROS compounds, leaves of all genotypes showed relatively light staining in the control samples. However, among the cold-treated group, leaves from WT plants were stained darker than those from transgenic plants (Figure 4A,B). These qualitative assessments were supported by quantitative measurements; O_2_^−^ levels were approximately 1.2–1.9 times higher in the WT than in the transgenic lines under cold stress conditions, and cold-stressed WT plants had approximately 1.2–2.1 times more H_2_O_2_ levels than the transgenic plants (Figure 4C,D). To assess the extent of membrane damage, which is associated with excess ROS, we measured MDA levels and electrolyte leakage. Both parameters showed similar values between the transgenic and WT plants under control conditions. In response to cold stress, MDA levels and electrolyte leakage were significantly reduced in transgenic plants compared to the WT (Figure 4E,F).

### 2.5. MsATG13 Increased Antioxidant Enzymes Activity and Proline Content under Cold Stress

**Figure 5:**
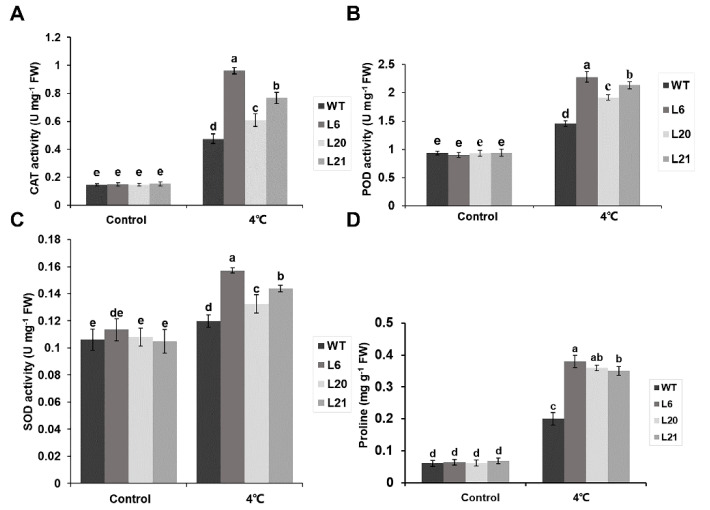
Antioxidant enzyme activity and proline content. (**A**–**C**) Activities of (**A**) catalase (CAT), (**B**) peroxidase (POD), and (**C**) superoxide dismutase (SOD) in the leaves of WT and transgenic tobacco plants incubated at 4 °C. (**D**) Proline content in the leaves of WT and transgenic tobacco plants incubated at 4 °C. Data are presented as the mean ± standard deviation from three biological replicates. Letters above each bar indicate statistical significance groups at *p* < 0.05 (one–way analysis of variance).

To identify a potential mechanism by which *MsATG13* expression may have reduced oxidative damage, we assessed the activities of three key antioxidant enzymes: SOD, POD, and CAT. Activities of all three were increased under cold stress conditions compared to the control plants. Furthermore, all three enzymes showed higher activity in the transgenic plants than in WT plants. Specifically, in the cold–stressed group, CAT and POD activities were approximately 1.3–2.0 times higher among transgenic plants than WT plants (Figure 5A,B). SOD activity was also significantly higher in the transgenic lines than that in WT plants (Figure 5C). Finally, we assessed the effects of *MsATG13* overexpression on the non–enzymatic antioxidant system, namely proline content. In control plants, proline levels were similar between the WT and the transgenic lines. However, among cold-treated plants, the transgenic lines had significantly higher proline content than WT plants (~1.7–1.9 times higher) (Figure 5D). These results suggested that *MsATG13* overexpression reduced oxidative damage in response to cold stress by influencing both the enzymatic and non–enzymatic antioxidant systems.

## 3. Discussion

Autophagy is a conserved intracellular degradation pathway that is crucial to plant survival in environmental stress conditions [32,33]. In the present study, we characterized the role of a *M. sativa* gene associated with autophagy, *MsATG13*, in the cold stress response; the survival of alfalfa under cold stress depends on the crown buds [34] and this gene was previously shown to be upregulated in the crown buds of a naturally cold–tolerant alfalfa cultivar during cold exposure. Initial expression profiling showed that *MsATG13* was upregulated in both the leaves and the roots in response to cold treatment and it was much more strongly induced in the leaves (Figure 1C). This is consistent with earlier studies demonstrating the important role of autophagy in maintaining organellar function and metabolite homeostasis in the leaves [35,36].

To further characterize this gene, we overexpressed it in tobacco. In response to cold stress, transgenic tobacco plants showed less damage than WT plants (Figure 2C). Furthermore, cold–stressed transgenic plants contained significantly more autophagosomes than WT plants (Figure 3A), indicating that *MsATG13* overexpression increased levels of autophagy in cold–stressed tobacco plants. Similar phenomena have been observed in previous studies; for example, transgenic apples overexpressing *MdATG8i* have more autophagosomes and increased salt stress tolerance compared to WT apples [37].

The key steps of autophagosome production are initiation, nucleation, membrane extension, and maturation. *ATG13* reportedly mediates the initiation step, and overexpression of this gene may also promote expression of other ATGs. Indeed, we here found that exogenous overexpression of *MsATG13* in cold–stressed tobacco plants triggered upregulation of the endogenous ATGs. *ATG1* serves as a crucial constituent of the upstream *ATG13–ATG1* complex, which also plays a critical role in the initiation of autophagosomes formation [38]. In this study, it was found that the expression level of *NtATG1* in transgenic lines was significantly higher than that of WT under cold stress conditions (Figure 3C). *ATG9* is an important membrane source for inducing the membrane elongation of autophagosomes [39], *ATG6* is responsible for the nucleation of autophagosomes [40], and we found that *MsATG13* overexpression significantly increased the expression levels of *NtATG6* and *NtATG9* in transgenic lines compared with WT under cold stress (Figure 3D,F). *ATG8* is an important member of the ubiquitin–like conjugation systems which are responsible for the maturation of autophagosomes [41]. Additionally, *NtATG8* was upregulated in both the transgenic lines and WT, showing higher expression level in the transgenic lines under cold stress (Figure 3E). There was no significant difference in the expression levels of the four NtATGs genes of all plants under control conditions, and their expression was induced by cold stress. After cold stress, their expression levels were significantly higher in transgenic tobacco than in WT, which is consistent with the changes in the number of autophagosomes. The *MsATG13* overexpression promoted the expression level of other key NtATGs which are necessary for the production of autophagosomes, which therefore promoted the formation of more autophagosomes in transgenic lines, enhancing the level of autophagy and thus participating in the regulation of cold stress tolerance.

Autophagy appears to be a necessary process by which cells clear excess ROS [42]. The primary forms of ROS accumulated by cold–stressed plants are O_2_^−^ and H_2_O_2_ [43]. Accumulation of compounds such as these can lead to membrane damage via lipid peroxidation [44]. MDA and electrolyte leakage are two commonly–used proxies used to evaluate abiotic stress tolerance based on the degree of membrane damage [45,46]. We here found that *MsATG13*–overexpressing tobacco plants, which had higher autophagy levels, accumulated lower levels of ROS compared with WT plants (Figure 4A–D). Furthermore, MDA contents and electrolyte leakage levels of *MsATG13* transgenic tobacco were significantly lower than those of WT (Figure 4E,F), indicating decreased oxidative damage among transgenic plants.

Autophagy may also improve the antioxidant system by regulating the activity of antioxidant enzymes [15]. *MsATG13* transgenic tobacco, which had higher autophagy levels than WT plants, also showed higher CAT, POD, and SOD activities in response to cold stress (Figure 5A–C). The non–enzymatic antioxidant system includes proline, accumulation of which improves cold stress tolerance in plant cells by stabilizing osmotic resistance, increasing cell turgor, and controlling water consumption [47]. In addition to acting as an osmotic protector, proline also plays an important role in ROS scavenging and the promotion of antioxidant enzyme activity [48]. Here, proline levels were shown to be significantly higher in transgenic tobacco than WT under cold stress conditions (Figure 5D). This indicated that *MsATG13* overexpression improved the antioxidant system (perhaps via the enhanced autophagy levels mediated by *MsATG13*), promoting ROS degradation in response to cold stress.

In summary, we here studied the roles of *MsATG13* in mediating autophagy and regulating plant tolerance to cold stress. Our results showed that *MsATG13* was shown to promote autophagy by upregulating other key ATGs necessary for autophagosome production, which may have also enhanced the antioxidant system. Enhanced antioxidant levels served to reduce ROS accumulation in response to cold treatment, preventing cellular damage due to excess ROS. Overall, these results demonstrated that *MsATG13* plays a significant role in enhancing plant cold tolerance through its regulation of autophagy, which is inseparable from its association with other autophagy related genes. *MsATG13* regulates autophagy by influencing other autophagy–related genes, thereby enhancing the cold tolerance of plants. However, the molecular mechanisms involved in this process require further investigation. *MsATG13* regulates autophagy by influencing other autophagy–related genes, thereby enhancing the cold tolerance of plants. However, the molecular mechanisms involved in this process require further investigation. This study increases our understanding of the role that autophagy plays in promoting plant cold tolerance. Furthermore, identification of *MsATG13* as a critical regulator of resistance to cold stress and offers future opportunities for breeding plants with cold tolerance.

## 4. Materials and Methods

### 4.1. Plant Materials

*M. sativa* ‘Zhaodong’ seeds were provided by the Institute of Animal Husbandry, Heilongjiang Province, China. The seeds were placed in culture dishes containing distilled water and incubated at 18 °C in the dark for 48 h to sprout. The sprouted seeds were transferred to jars containing vermiculite and ½× Hoagland solution and were incubated under a 14/10 h light/dark photoperiod (24/18 °C) with 60–80% relative humidity.

Tobacco seeds (*Nicotiana tabacum*) were disinfected with alcohol and sodium hypochlorite and placed on culture dishes containing Murashige and Skoog [49] medium (pH 5.8). The plates were incubated under the conditions described above for the sprouted *M. sativa* seedlings. After 1 week, germinated seedlings were transplanted into culture flasks containing Murashige and Skoog medium, then grown under the same conditions. After another 3 weeks, the seedlings were transferred to jars containing vermiculite and ½× Hoagland solution, then returned to the growth chamber until they were collected for cold tolerance assessments.

### 4.2. Gene Cloning and Sequence Analysis

Total RNA was isolated from *M. sativa* crown buds using the RNAprep Pure Plant Kit (TianGen Biotech, DP432, Beijing, China). cDNA was synthesized using One–Step gDNA Removal and cDNA Synthesis SuperMix (TransGen Biotech, AE311, Beijing, China). The coding sequence (CDS) of *MsATG13* (MW774897.1) was amplified from alfalfa cDNA using primer pair P1 (Supplementary Table S1) and Ex Taq DNA Polymerase (TaKaRa Biotech, RR001B, Beijing, China). The PCR amplification procedure was as follows: initial denaturation for 5 min at 95 °C; 35 cycles of 30 s at 95 °C, 30 s at 50 °C, and 2 min at 72 °C; and final extension for 10 min at 72 °C. The *MsATG13* CDS was inserted into the pMD18–T vector for subsequent analysis with sequencing. The open reading frame (ORF) and amino acid sequence of MsATG13 (UUH54286.1) were downloaded from NCBI: https://www.ncbi.nlm.nih.gov/orffinder (accessed on 20 April 2023). Amino acid domains of MsATG13 were identified with SMART: http://smart.embl-heidelberg.de (accessed on 20 April 2023) and the specific type and function description of the domains were searched with InterPro: http://www.ebi.ac.uk/interpro (accessed on 17 July 2023). Relevant amino acid sequences were downloaded from NCBI: https://blast.ncbi.nlm.nih.gov/Blast.cgi (accessed on 20 April 2023) and these sequences were aligned with ClustalW and a phylogenetic tree was constructed in MEGA 5.0 [50] using the neighbor–joining (NJ) method with 1000 bootstrap replicates.

### 4.3. Gene Expression Analysis

Four–weeks–old alfalfa seedlings were incubated at 4 °C and the leaves and roots were collected at 0, 1, 3, 6, 12, and 24 h. RNA was extracted and cDNA was synthesized as described above. Expression levels of *MsATG13* were then analyzed with quantitative reverse transcription (qRT)–PCR using the TransStart Tip Green qPCR SuperMix (TransGen Biotech, AQ141, Beijing, China) and the primer pair P2 (Supplementary Table S1). Reactions were carried out on a 7300 Real–Time PCR System (Applied Biosystems, Waltham, MA, USA) with the following program: initial denaturation for 30 s at 94 °C, 40 cycles of 5 s at 94 °C, 31 s at 54 °C, and 31 s at 72 °C. Average gene expression levels were calculated from three technical replicates of each sample. The relative expressing levels were determined on the basis of the 2^−ΔΔCt^ method [51], and the *MsActin2* (JQ028730.1) was used as the internal control gene. All data were normalized to the expression level in the control (0 h). There were three independent replicates of this experiment.

### 4.4. Production of Transgenic Tobacco Lines and Cold Tolerance Assessments

An expression vector containing the cauliflower mosaic virus (CaMV) 35S promoter [52] was digested with *Sac* I and *Pst* I, then the *MsATG13* CDS was inserted to produce the pCaMV35S–*MsATG13* construct; this included the bar gene, encoding phosphinothricin acetyltransferase, to allow transformant screening. The *Agrobacterium tumefaciens* strain EHA105 was transformed with the resulting construct using the freeze–thaw method. Transgenic tobacco plants were generated with the *Agrobacterium*–mediated leaf disc transformation method [53]. Transformants were verified through PCR with primer pair P3 (Supplementary Table S1) and EasyTaq DNA Polymerase (TransGen Biotech, AP111, Beijing, China). The PCR amplification procedure was as follows: initial denaturation for 5 min at 95 °C; 30 cycles of 30 s at 95 °C, 30 s at 52 °C, and 40 s at 72 °C; and final extension for 10 min at 72 °C. *MsATG13* expression was quantified in the transgenic and WT plants via qRT–PCR with the primer pair P2 and the following program: initial denaturation for 30 s at 94 °C, 40 cycles of 5 s at 94 °C, 31 s at 54 °C, and 31 s at 72 °C. Average gene expression levels were calculated from three technical replicates of each sample. The relative expressing levels were determined on the basis of the 2^−ΔΔCt^ method, and *NtGAPDH* (XM_016655379.1) was used as the internal control gene. All data were normalized to that in the WT plant. There were three independent replicates of this experiment.

To assess the effects of *MsATG13* overexpression on cold tolerance, transgenic and WT tobacco plants were raised to the six–weeks stage, then transferred to an artificial climate incubator. Plants were incubated at 4 °C for 4 h, followed by −4 °C for 3 h [54]. Control plants were maintained at 25 °C for all 7 h. A visual assessment of plant growth was used to determine cold tolerance.

### 4.5. Analyses of Oxidative Damage in Cold–Treated Plants

#### 4.5.1. Histochemical Staining

WT and transgenic tobacco plants were raised to the six–weeks stage, then incubated at 4 °C (cold–stress group) or 25 °C (control group) for 24 h, then leaves were collected. Levels of superoxide anion radicals (O_2_^−^) and hydrogen peroxide (H_2_O_2_) were measured via histochemical staining with p-Nitro blue tetrazolium chloride (NBT) and diaminobenzidine (DAB), respectively, as described by [55] (with slight modifications). Leaves were fully immersed in NBT dye solution, vacuum infiltrated for 10 min, then incubated for 24 h in the dark at room temperature. After the dye solution was discarded, leaves were decolorized for 15 min in a rinse solution (3:1:1 ethanol:glycerol:lactic acid) in a boiling water bath. After the samples cooled, the rinse solution was replaced, and samples were incubated for another 15 min in the boiling water bath. After samples once again cooled, the rinse solution was replaced and samples were incubated in the dark at room temperature for 1–2 h. After chlorophyll decolorization was complete, the leaves were imaged. DAB staining was performed using similar methods, using DAB dye solution and a rinse solution (3:1:1 ethanol:acetic acid:glycerol).

#### 4.5.2. ROS Quantification

O_2_^−^ and H_2_O_2_ contents were next measured to quantify ROS levels. The O_2_^−^ content was measured as described by [56]. Briefly, leaves were ground in pre–cooled phosphate buffer (pH 7.8). Samples were centrifuged, then the supernatant was removed and mixed with hydroxylamine hydrochloride and incubated for 1 h at 25 °C. P–amino benzene sulfonic acid and a–naphthylamine were added and samples were incubated at 30 °C for 30 min. Finally, absorbance was measured at 530 nm to calculate the O_2_^−^ content.

H_2_O_2_ content was determined using the titanium sulfate method [57] with slight modifications. Leaves were ground in pre–cooled acetone and centrifuged. The resulting supernatant was mixed well with titanium sulfate and concentrated ammonia, then centrifuged. The supernatant was discarded, and the pellet was fully dissolved in sulfuric acid. Absorbance was then measured at 405 nm and the H_2_O_2_ content was calculated.

#### 4.5.3. Electrolyte Leakage Measurement

Electrolyte leakage was measured [58] with slight modifications. Tobacco leaf discs were placed into 20 mL deionized water, vacuum infiltrated for 15 min, then incubated at room temperature for 20 min. Initial conductivity (C1) was measured using a conductivity meter. The leaf discs were then boiled for 20 min and cooled to 25 °C before conductivity was measured again (C2). C1 and C2 were used to calculate electrolyte leakage.

#### 4.5.4. Malondialdehyde (MDA) Measurement

MDA was measured [59] with some modifications. Briefly, leaves were ground in trichloroacetic acid, then centrifuged. The supernatant was mixed with thiobarbituric acid and incubated in a boiling water bath for 15 min. After cooling to room temperature, the absorbance at wavelengths of 532 and 450 nm were measured to calculate MDA content.

### 4.6. Proline Content and Antioxidant Enzyme Activity Assays

Leaves were collected from the tobaccos of cold–treated and control groups. Proline was measured [60] with slight modifications. Leaves were ground in sulfosalicylic acid and incubated in a boiling water bath for 10 min, then centrifuged. The supernatant was mixed with glacial acetic acid and acidic ninhydrin, then incubated in a boiling water bath for 15 min. Samples were cooled to room temperature, then mixed with toluene. After standing and layering, the absorbance of the upper red extraction solution was measured at 520 nm to calculate proline contents.

The activities of SOD, CAT, and POD were measured as described by [61,62,63], respectively, with slight modifications. Leaves were ground in pre–cooled phosphate buffer (pH 7.8) and centrifuged to obtain the supernatant. To measure SOD activity, the supernatant was mixed with a SOD reaction solution (composed of methionine, EDTA–Na2, NBT, riboflavin, and phosphate buffer at pH 7.8). The reaction was allowed to proceed under illumination for 25 min, and the absorbance at the wavelength of 560 nm was measured to calculate SOD activity. To measure CAT activity, the supernatant described above was mixed with H_2_O_2_ and the absorbance at 240 nm was measured to calculate CAT activity. For the detection of the POD activity, leaves were ground in pre–cooled phosphate buffer (pH 6.0) and centrifuged to obtain the supernatant. The supernatant was mixed with POD reaction solution (composed of guaiacol, H_2_O_2_, and phosphate buffer at pH 6.0) and the change in absorbance at the wavelength of 470 nm was measured to calculate POD activity.

### 4.7. Autophagosome Quantification

Autophagosomes were observed as previously described [64]. Briefly, leaves were collected from the cold–treated and control plants, immediately cut into small pieces, and fixed with 2.5% glutaraldehyde in the dark for 12 h at 4 °C. After washing with phosphate buffer (pH 7.4), leaf fragments were fixed for 2.5 h in 1% (*v/v*) osmium tetroxide, then dehydrated in a graded ethanol series and embedded in Epon 812. Ultrathin sections (50–80 nm) were prepared with an EM UC7 ultramicrotome (Leica, Wetzlar, Germany) and collected on grids. The sections were visualized via transmission electron microscopy (TEM) on an H–7650 microscope (Hitachi, Tokyo, Japan) to observe and count the autophagosomes. 

### 4.8. Autophagy-Related Genes Expression Analysis

Leaves were collected from tobaccos of cold–treated and control groups. Total RNA was isolated, and cDNA was synthesized as described above. Expression levels of autophagy–related genes *NtATG1* (KR336558.1), *NtATG6* (KP316403.1), *NtATG8* (KR336564.1), and *NtATG9* (KR336569.1) were measured with qRT–PCR. The thermal cycling program was as follows: initial denaturation for 30 s at 94 °C, 40 cycles of 5 s at 94 °C, 31 s at 54 °C, and 31 s at 72 °C. Average gene expression levels were calculated from three technical replicates of each sample. The relative expressing levels were determined on the basis of the 2^−ΔΔCt^ method, *NtGAPDH* (XM_016655379.1) was used as the internal control gene. All data were normalized to the expression level in WT of the controls, respectively. There were three independent replicates of this experiment. Primers for each gene are listed in Supplementary Table S1.

### 4.9. Statistical Analysis

There were three independent replicates of each experiment, from which the mean values and standard deviations were calculated. Significant differences between pairs of samples were analyzed with Student’s *t*-test (* *p* < 0.05, ** *p* < 0.01, *** *p* < 0.001). Differences between three or more samples were assessed with one–way analysis of variance (ANOVA, *p* < 0.05).

## Data Availability

The data presented in this study are available on request from the corresponding author.

## Supplementary Materials

The following supporting information can be downloaded at: https://www.mdpi.com/article/10.3390/ijms241512033/s1.
