# Supplementary material for: Alfalfa MsATG13 Confers Cold Stress Tolerance to Plants by Promoting Autophagy
Source: Int J Mol Sci. 2023 Jul 27;24(15):12033. doi: 10.3390/ijms241512033 (PMC10418659; doi:10.3390/ijms241512033)
Supplement: Supplementary file 1 [file ijms-24-12033-s001.zip › ijms-2497866-supplementary.doc]

**Supplementary Table S1 Primers used in this study.**

| **Pair name** |  | **Primer orientation** | **Sequence (5′–3′)** | **Experiment** |
| --- | --- | --- | --- | --- |
| P1 |  | F | CGAAAAGGTTTTGATAGTTG  TCACCATTCCAAACATAGC | *MsATG13* cloning |
|  |  | R |  |  |
| P2 |  | F | CGTCCCTTTACCCCTGCTC  TGGAGATGCCAATGGACTTC | qRT-PCR |
|  |  | R |  |  |
| P3 |  | F | TTTGCTTTTGAGGTCTTTGT  TGGGGATCATCTTTTCTTAG | Transgenic plant verifying |
|  |  | R |  |  |
| *MsActin2* |  | F | TGGTTCAACTATGTTTCCAG  TCCATAACATGACAGAGACAC | qRT-PCR |
|  |  | R |  |  |
| *NtGAPDH* |  | F | CTTTTTGGTGAGAAGTCCGT  ACAGTAGTCATGAGACCCTC | qRT-PCR |
|  |  | R |  |  |
| *NtATG1* |  | F | CCTTTCCACGGTAGGACTAG  GCTTCGCCTATCTTCAACAA | qRT-PCR |
|  |  | R |  |  |
| *NtATG6* |  | F | ATGCTCCTACCCAGCCTAAC  CTTCAGAAAATCAGCCTCAC | qRT-PCR |
|  |  | R |  |  |
| *NtATG8* |  | F | GGGAGAAGTATCCTGATAGAA  TTCCTCATAAATCGCAGACA | qRT-PCR |
|  |  | R |  |  |
| *NtATG9* |  | F | TGATGTTACTTGAGGAGAT  GGAAGGGTAGCTATTTAGAA | qRT-PCR |
|  |  | R |  |  |

F, forward; R, reverse; qRT-PCR, quantitative reverse transcription PCR; *Ms*, *Medicago sativa*; *Nt*, *Nicotiana tabacum*.
